# Comprehensive evaluation of corneas from normal, forme fruste keratoconus and clinical keratoconus patients using morphological and biomechanical properties

**DOI:** 10.1007/s10792-020-01679-9

**Published:** 2021-01-03

**Authors:** Hui Zhang, Lei Tian, Lili Guo, Xiao Qin, Di Zhang, Lin Li, Ying Jie, Haixia Zhang

**Affiliations:** 1 School of Biomedical Engineering, Capital Medical University, Beijing, 100069 China; 2 Beijing Institute of Ophthalmology, Beijing Tongren Eye Center, Beijing Tongren Hospital, Capital Medical University and Beijing Ophthalmology & Visual Sciences Key Laboratory, Beijing, 100730 China; 3 Beijing Key Laboratory of Fundamental Research On Biomechanics in Clinical Application, Capital Medical University, Beijing, 100069 China; 4 Beijing Advanced Innovation Center for Big Data-Based Precision Medicine, Beihang University & Capital Medical University, Beijing Tongren Hospital, Beijing, 100730 China

**Keywords:** Keratoconus, Forme fruste keratoconus, Morphology, Biomechanics

## Abstract

**Objective:**

To more comprehensively evaluate the ability of the parameters reflecting the morphological and biomechanical properties of the cornea to distinguish clinical keratoconus (CKC) and forme fruste keratoconus (FFKC) from normal.

**Methods:**

Normal eyes (*n* = 50), CKC (*n* = 45) and FFKC (*n* = 15) were analyzed using Pentacam, Corvis ST and ORA. Stepwise logistic regression of all parameters was performed to obtain the optimal combination model capable of distinguishing CKC, FFKC from normal, named SLR1 and SLR2, respectively. Receiver operating characteristic (ROC) curves were applied to determine the predictive accuracy of the parameters and the two combination models, as described by the area under the curve (AUC). AUCs were compared using the DeLong method.

**Results:**

The SLR1 model included only the TBI output by Pentacam, while the SLR2 model included the morphological parameter F.Ele.Th and two parameters from the Corvis ST, HC DfA and SP-A1. The majority of the parameters had sufficient strength to differentiate the CKC from normal corneas, even the seven separate parameters and the SLR1 model had a discrimination efficiency of 100%. The predictive accuracy of the parameters was moderate for FFKC, and the SLR2 model (0.965) presented an excellent AUC, followed by TBI, F.Ele.Th and BAD-D.

**Conclusion:**

The F.Ele.Th from Pentacam was the most sensitive morphological parameter for FFKC, and the combination of F.Ele.Th, HC DfA and SP-A1 made the diagnosis of FFKC more efficient. The CRF and CH output by ORA did not improve the combined diagnosis, despite the corneal combination of morphological and biomechanical properties that optimized the diagnosis of FFKC.

## Introduction

Keratoconus is a non-inflammatory cone-like ectasia of the cornea, which is usually bilateral and progresses over time [1, 2]. Despite extensive research in this field, the exact etiology of the disease is not clear, and the current diagnostic criteria for the disease are mainly based on the combination of symptoms and signs of the disease as well as morphological evaluation of the cornea [3].

Recently, some researchers [4, 5] have proposed that during the progression of keratoconus, the corneal Bowman’s membrane ruptures, resulting in the disorganization of the collagen fibers and imbalance of its material composition, which further caused the corneal protein kinase and other catabolic enzyme levels to increase and the protein kinase inhibitor levels to decrease. These enzyme changes destroy the collagen structure of the cornea and reduce the corneal stroma, which leads to unstable corneal biomechanical properties and reduced corneal mechanical strength, thus causing corneal thinning and ectasia. Thus, the changes in corneal biomechanical properties may precede the morphological changes as keratoconus progresses. Therefore, current research is focused on evaluating the biomechanical characteristics or the combined morphological and biomechanical characteristics of the disease.

The Ocular Response Analyzer (ORA) and Corneal Visualization Scheimpflug Technology (Corvis ST) are the two most recognized devices that are used to measure corneal biomechanics in vivo. Herber et al. [6] have proposed that both devices are appropriate to distinguish healthy eyes from keratoconic eyes with high sensitivity and specificity, even though the ability of ORA to identify keratoconus was less than for Corvis ST. Also, for Pentacam, which currently is the most widely used morphological detection device in clinics, most of the studies [7–9] agreed that Pentacam was comparable to Corvis ST with respect to its ability to distinguish keratoconus from normal corneas. And the tomographic and biomechanical index (TBI), which is the combined parameters of the two devices, had the highest diagnostic capability for keratoconus. However, for Pentacam and ORA, some studies [10, 11] suggested that the discriminating ability of the corneal hysteresis (CH) and corneal resistance factor (CRF) from ORA was poor, although most of the other parameters of the output of these two devices were significantly different between the keratoconus and normal eyes. Furthermore, although a few studies have used the above three kinds of devices for analysis, they were all studies on patients with clinical keratoconus [5, 12, 13]. And at most, these studies only analyzed and compared the diagnostic ability of single output parameters of the devices [5, 12].

In the present study, we mainly intend to evaluate the ability of the parameters reflecting the morphological and biomechanical properties of the cornea to distinguish clinical keratoconus (CKC) and forme fruste keratoconus (FFKC) from normal more comprehensively, so as to further explore three devices that are described above concerning the value of their output parameters in the diagnosis of early keratoconus.

## Materials and methods

### Subject recruitment

This prospective comparative study included patients with clinical keratoconus, forme fruste keratoconus and candidates for refractive surgery with normal corneas, who served as the control group. A diagnosis of clinical keratoconus (CKC group) was made if the eye met the following criteria [14, 15], (1) an irregular cornea as determined by distorted keratometry mires, distortion of the retinoscopic or ophthalmoscopic red reflex (or a combination of the two) and (2) at least one of the following biomicroscopic signs, Vogt’s striae, Fleischer’s ring of < 2 mm arc or corneal scarring consistent with keratoconus. An eye was diagnosed as having forme fruste keratoconus (FFKC group) if it was the fellow eye of a patient with keratoconus and showed the following features [3, 16]: (1) a normal-appearing cornea on slit-lamp examination, retinoscopy and ophthalmoscopy, (2) normal topography with no asymmetric bowtie and no focal or inferior steepening pattern, (3) the level of topographic keratoconus classification (TKC) provided by Pentacam was normal, namely, it was “-,” and (4) the patient had no history of contact lens use, ocular surgery or trauma. For candidates undergoing refractive surgery, only one eye from each person was chosen using a random numbers table. Exclusion criteria included a history of corneal or ocular surgery, significant corneal scarring and significant ophthalmic disease that might potentially affect the study outcome.

Patients who wore contact lens were asked to remove soft contact lenses at least two weeks and rigid contact lenses at least one month before assessment. Data were collected from August 2019 to January 2020 from the Beijing Tongren Hospital, Capital Medical University. All participants signed an informed consent form in accordance with the tenets of the Declaration of Helsinki. This study was approved by the Ethics Committee of the Beijing Tongren Hospital, Beijing, China.

### Ocular examination

A comprehensive ocular examination was performed on the eyes of all subjects, including a detailed assessment of uncorrected distance visual acuity, corrected distance visual acuity, slit-lamp microscopy, fundus examination, tomography measurements using Scheimpflug imaging (Pentacam; Oculus, Optikgeräte GmbH, Wetzlar, Germany), biomechanical examination using the Corvis ST (Oculus; Optikgeräte GmbH, Wetzlar, Germany) and ORA (Reichert Ophthalmic Instruments; Buffalo, NY, USA). All measurements were taken between 09:00 and 17:00 on the same day and by the same trained ophthalmologists.

### Pentacam measurement

The Pentacam (software version 1.20r134) reconstructs a three-dimensional image of the entire anterior segment of the eye from the anterior surface of the cornea to the posterior surface of the lens by utilizing the high-speed rotating Scheimpflug system. Details and principles of the Pentacam are described elsewhere [17]. Only scans that the Pentacam “quality specification” (QS) function determined as “OK” are included for analysis.

Parameters included in the analysis were the index of surface variance (ISV), index of vertical asymmetry (IVA), keratoconus index (KI), central keratoconus index (CKI), index of height asymmetry (IHA), index of height decentration (IHD), maximum keratometry from the anterior corneal surface (Kmax F), maximum Ambrósio relational thickness (ARTmax), inferior–superior difference value (I-S), Belin–Ambrósio enhanced ectasia total deviation index (BAD-D), the elevation of the front surface at the thinnest location (F.Ele.Th), the elevation of the back surface at the thinnest location (B.Ele.Th), mean keratometry from the anterior corneal surface (Km F), central astigmatism from the anterior corneal surface (Astig F), corneal thickness at the apex of the cornea (CCT), pachymetry at the thinnest point (TP) and corneal volume at 10 centered at the thinnest point (CV 10).

### Corvis ST measurement

The Corvis ST (software version 1.5r1902) evaluates the dynamic corneal deformation response to an air-puff pulse. Details and principles of the Corvis ST are described elsewhere [5]. Only measurements where the “quality specification” read OK were accepted. If the comment was marked as yellow or red, the examination was repeated. The following parameters were included for analysis: maximum deformation amplitude at the corneal apex (DA), the first applanation time (A1T), the first velocity of applanation (A1V), the second applanation time (A2T), the second velocity of applanation (A2V), time from the start until the highest concavity (HCT), peak distance (PD), central curvature radius at the highest concavity (Radius), Ambrósio relational thickness to the horizontal profile (ARTh), the biomechanical-corrected intraocular pressure (bIOP), stiffness parameter at the first applanation (SP-A1), the deflection length of the first (A1 DfL) and second applanation (A2 DfL) as well as the highest concavity (HC DfL), the deflection amplitude of the first (A1 DfA) and second applanation (A2 DfA) as well as the highest concavity (HC DfA), the maximal value of the ratio between the deformation amplitude at the apex and at 1 (DA Ratio 1) and 2 mm (DA Ratio 2) from the corneal apex, the Corvis biomechanical index (CBI) and TBI.

### ORA measurement

The ORA (software version 4.12) measures the deformation of the cornea by an air-puff tonometer in response to a 20-ms jet of air, using a bidirectional applanation process. Details and principles of the ORA are described elsewhere [18]. Measurements were repeated until the signal score was > 4.0. Then the values of CH and CRF were recorded and included in the analysis.

### Statistical methods

Statistical analysis was performed using SPSS version 20.0 (SPSS Inc., Chicago, IL, USA) and MedCalc software version 19.1 (MedCalc Software bvba, Ostend, Belgium). The Chi-square test was performed to describe the gender differences among the groups. The Shapiro–Wilk test was used to check for normal distribution of quantitative data. For the two groups of data conforming to the normal distribution, which were here provided as the mean and standard deviation (SD), the differences between the two groups were analyzed using the independent sample *t* test. For data that did not conform to the normal distribution, they were expressed with median and range of variation, and the differences between groups were tested by the Mann–Whitney *U* test.

All the included parameters were analyzed using logistic regression with forward stepwise inclusion to determine the optimal combination model capable of distinguishing clinical keratoconus (CKC group) and forme fruste keratoconus (FFKC group) from normal corneas (control group), respectively. Receiver operating characteristic (ROC) curves were applied to determine the predictive accuracy of the parameters and the combination models, as described by the area under the curve (AUC). An area of 1.0 represented a perfect test, while an area of 0.5 represented an ineffective test. The diagnostic specificity and sensitivity of all individual parameters with the highest AUC were evaluated, and cutoff values were determined. Also, AUCs were compared using the nonparametric DeLong method. A *p-*value < 0.05 was considered to be statistically significant.

## Results

Forty-five eyes from 30 patients (20 males and 10 females, and a mean age of 26.06 ± 6.04 years) were included in the CKC group. Among the patients in the CKC group, both eyes of 15 patients were included because they presented bilateral keratoconus. The remaining 15 patients had unilateral disease. The normal contralateral eyes of the patients in the CKC group with unilateral keratoconus constituted the FFKC group (11 males and 4 females, and a mean age of 27.04 ± 7.68 years). The control group consisted of 50 normal individuals (36 males and 14 females, and a mean age of 28.18 ± 5.71 years). Only one eye per person was randomly evaluated in the control group. There were no statistically significant differences between the groups in age or sex distribution (*p* > 0.05).

Except for HCT, PD, A1 DfL and A2 DfL, statistically significant differences were found in the parameters between the CKC and control groups (*p* < 0.001) (Table 1). Similarly, except for HCT, PD, HC DfL and A2 DfL, the other parameters in the FFKC group were significantly different from the CKC group (*p* < 0.001). There also were statistical differences in more than half of the parameters between the FFKC and control groups (*p* < 0.05). In addition, F.Ele.Th and TBI were the only parameters with significant statistical differences when any two groups of the three groups were compared (*p* < 0.001).

**Table 1:** Comparison of corneal morphological and biomechanical parameters by groups

Parameters	CKC group (*n* = 45)	FFKC group (*n* = 15)	Control group (*n* = 50)	CKC group versus control group (*p* value)	FFKC group versus control group (*p* value)	CKC group versus FFKC group (*p* value)
*Morphological parameters (Pentacam’s output parameters)*						
BAD-D	10.37 (3.38–27.85)	2.14 ± 1.02	0.99 ± 0.58	< 0.001^b^	0.001^a^	< 0.001^b^
F.Ele.Th (μm)	22 (− 3 to 68)	4.47 ± 1.55	2 (0–5)	< 0.001^b^	< 0.001^b^	< 0.001^b^
B.Ele.Th (μm)	55 (19–250)	9.53 ± 5.94	4 (0–11)	< 0.001^b^	0.001^b^	< 0.001^b^
ARTmax	127.96 ± 59.71	339.53 ± 83.46	420.5 (315.0–602.0)	< 0.001^b^	0.001^b^	< 0.001^a^
I-S (D)	5.68 (− 2.31 to 16.98)	1.21 ± 0.95	0.36 ± 0.64	< 0.001^b^	0.004^a^	< 0.001^b^
Km F (D)	49.9 (39.7–72.5)	43.36 ± 1.78	43.20 ± 1.21	< 0.001^b^	0.751^a^	< 0.001^b^
Astig F (D)	4.01 ± 2.42	1.04 ± 0.74	1.30 ± 0.61	< 0.001^a^	0.221^a^	< 0.001^a^
CCT (μm)	450 (366–667)	515.73 ± 27.55	541.84 ± 31.82	< 0.001^b^	0.005^a^	< 0.001^b^
TP (μm)	441.71 ± 38.84	511.27 ± 26.39	538.3 ± 31.75	< 0.001^a^	0.003^a^	< 0.001^a^
CV 10 (mm^3^)	57.56 ± 2.87	59.60 ± 2.46	61.08 ± 3.48	< 0.001^a^	0.075^a^	0.015^a^
ISV	100 (37–202)	24.13 ± 7.04	18.06 ± 4.30	< 0.001^b^	0.006^a^	< 0.001^b^
IVA	0.90 (0.31–2.37)	0.18 (0.10–0.59)	0.13 (0.06–0.33)	< 0.001^b^	0.002^b^	< 0.001^b^
KI	1.23 (0.93–1.74)	1.05 ± 0.02	1.03 (0.93–1.10)	< 0.001^b^	0.002^b^	< 0.001^b^
CKI	1.09 (0.99–1.30)	1.01 (1.00–1.02)	1.01 (0.99–1.02)	< 0.001^b^	0.071^b^	< 0.001^b^
IHA	27.9 (0.2–86.7)	8.27 ± 5.09	6.1 (0.3–22.9)	< 0.001^b^	0.518^b^	< 0.001^b^
IHD	0.144 (0.039 t 0.385)	0.017 (0.007–0.059)	0.011 (0.004–0.03)	< 0.001^b^	0.005^b^	< 0.001^b^
KMax F (D)	58.1 (45.2–90.8)	44.97 ± 1.54	44.56 ± 1.38	< 0.001^b^	0.360^a^	< 0.001^b^
*Biomechanical parameters (Corvis ST’s output parameters)*						
DA (mm)	1.248 (0.943–1.850)	1.11 ± 0.10	1.12 ± 0.10	< 0.001^b^	0.750^a^	< 0.001^b^
A1T (ms)	6.91 ± 0.24	7.17 ± 0.21	7.246 (6.782–8.103)	< 0.001^b^	0.199^b^	< 0.001^a^
A1V (m/s)	0.177 (0.127–0.317)	0.16 ± 0.02	0.158 (0.104–0.191)	< 0.001^b^	0.821^b^	0.002^b^
A2T (ms)	22.23 ± 0.34	21.98 ± 0.3	21.95 ± 0.39	< 0.001^a^	0.823^a^	0.010^a^
A2V (m/s)	-0.344 (− 0.564 to − 0.209)	-0.29 ± 0.02	-0.285 (-0.351–-0.147)	< 0.001^b^	0.624^b^	< 0.001^b^
HCT (ms)	17.094 (15.939–17.787)	17.17 ± 0.49	17.094 (15.015–17.787)	0.227^b^	0.239^b^	0.768^b^
PD (mm)	5.195 (4.569–5.672)	5.19 ± 0.22	5.28 ± 0.25	0.113^b^	0.164^a^	0.608^b^
Radius (mm)	5.290 (2.611–9.040)	7.17 ± 0.95	7.529 (6.379–10.564)	< 0.001^b^	0.054^b^	< 0.001^b^
A1 DfL (mm)	2.367 (2.119–3.012)	(2.23–2.40)	2.376 (1.164–2.986)	0.160^b^	0.107^b^	0.026^b^
HC DfL (mm)	6.41 ± 0.44	6.59 ± 0.34	6.851 (4.425–7.700)	< 0.001^b^	0.031^b^	0.102^a^
A2 DfL (mm)	2.90 ± 0.64	2.89 ± 0.68	2.804 (1.834–4.411)	0.715^b^	0.474^b^	0.948^a^
A1 DfA (mm)	0.111 (0.089–0.188)	0.09 ± 0.01	0.099 (0.073–0.114)	< 0.001^b^	0.041^b^	< 0.001^b^
HC DfA (mm)	1.094 (0.825–1.717)	0.93 ± 0.08	0.96 ± 0.09	< 0.001^b^	0.270^a^	< 0.001^b^
A2 DfA (mm)	0.13 ± 0.02	0.11 ± 0.01	0.11 ± 0.01	< 0.001^a^	0.036^a^	< 0.001^a^
DA Ratio 1	1.70 ± 0.08	1.59 ± 0.05	1.556 (1.480–3.106)	< 0.001^b^	0.045^b^	< 0.001^a^
DA Ratio 2	6.176 (3.954–15.586)	4.61 ± 0.47	4.277 (3.587–6.022)	< 0.001^b^	0.029^b^	< 0.001^b^
ARTh	155.688 (10.466–479.813)	363.15 ± 98.78	446.06 ± 101.8	< 0.001^b^	0.009^a^	< 0.001^b^
bIOP (mmHg)	13.03 ± 2.33	14.50 ± 1.61	14.5 (10.8–22.7)	< 0.001^b^	0.858^b^	0.010^a^
SP-A1	48.51 ± 18.30	84.3 ± 15.05	95.51 ± 16.12	< 0.001^a^	0.020^a^	< 0.001^a^
CBI	1.000 (0.065–1.000)	0.731 (0.001–1.000)	0.068 (0.000–0.999)	< 0.001^b^	0.003^b^	< 0.001^b^
TBI	1.00 (0.99–1.00)	0.53 (0.01–1.00)	0.03 (0.00 0.91)	< 0.001^b^	< 0.001^b^	< 0.001^b^
*Biomechanical parameters (ORA’s output parameters)*						
CRF (mmHg)	6.98 ± 1.74	9.79 ± 0.88	11.0 (8.2–20.0)	< 0.001^b^	0.015^b^	< 0.001^a^
CH (mmHg)	8.17 ± 1.19	9.79 ± 1.01	10.7 (8.9–19.2)	< 0.001^b^	0.008^b^	< 0.001^a^

^a^Independent two-sample *t*-test

^b^Mann–Whitney *U* test

The two stepwise logistic regression models, named SLR1 and SLR2, were produced to distinguish the CKC and FFKC groups from the control group, respectively. Table 2 lists the details of these two combination regression models. Among them, the SLR1 combined model included only the TBI parameter output by Pentacam, while the SLR2 combined model consisted of the morphological parameter F.Ele.Th and the rest were output parameters from the Corvis ST, specifically, HC DfA and SP-A1.

**Table 2:** Specific information of the two stepwise logistic regression models of CKC group and FFKC group versus control group

	*β*	Standard error (SE)	Significance (*p*)
*SLR1 (CKC vs. control)*			
Constant	− 345.356	22,130.093	0.988
TBI	363.057	22,526.167	0.987
*SLR2 (FFKC vs. control)*			
Constant	53.838	19.820	0.007
F.Ele.Th	1.453	0.502	0.004
HC DfA	− 44.662	15.885	0.005
SP-A1	− 0.192	0.067	0.004

SLR1 = EXP(Beta1)/(1 + EXP(Beta1)) Beta1 =  − 345.356 + 363.057*TBI

SLR2 = EXP(Beta2)/(1 + EXP(Beta2)) Beta2 =  − 53.838 + 1.453* F.Ele.Th-44.662*HC DfA − 0.192*SP-A1

**Fig. 1:**
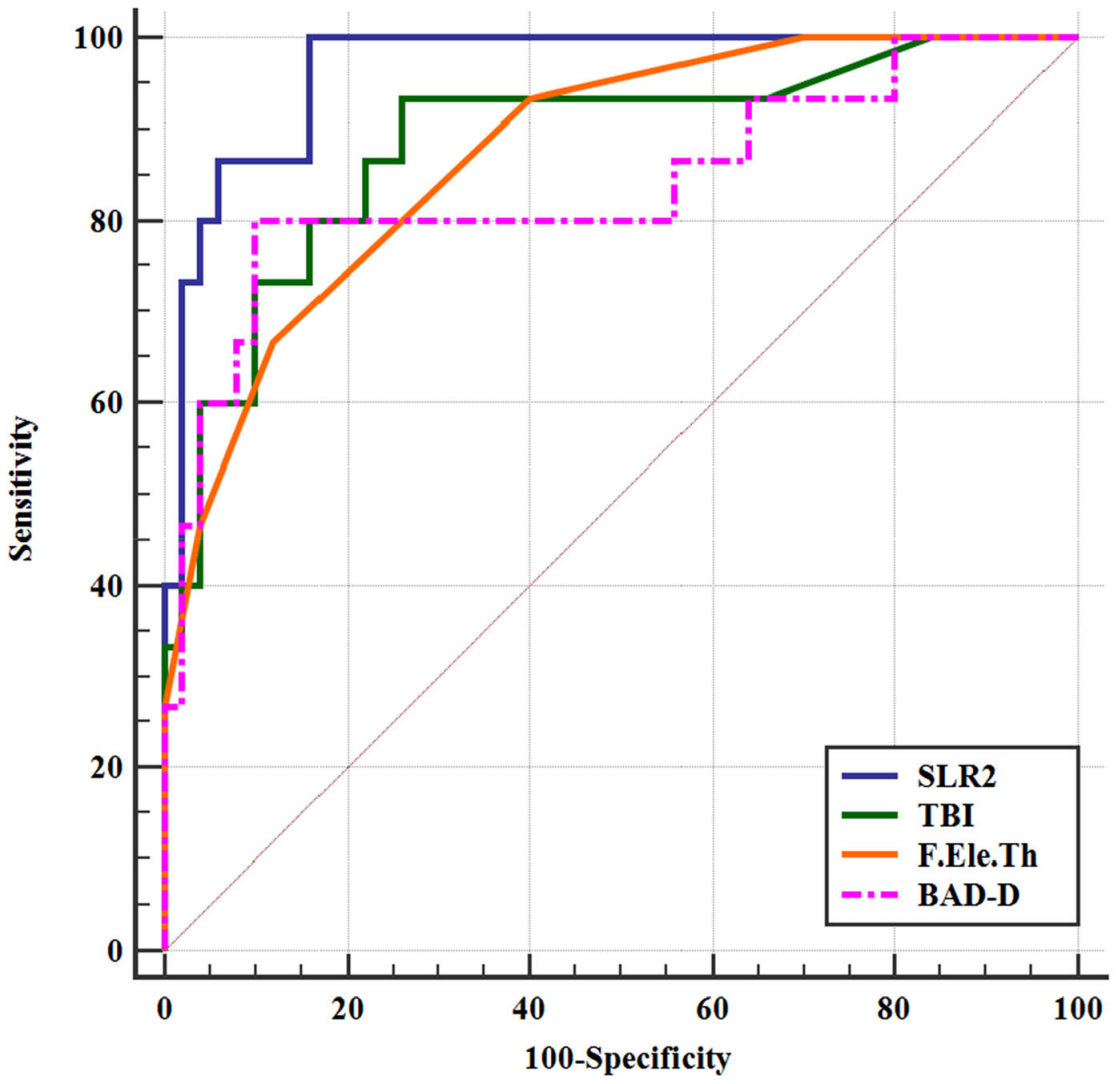
Receiver operating characteristic (ROC) curves for tomographic and biomechanical index (TBI), elevation of front surface in thinnest location (F.Ele.Th), Belin–Ambrósio enhanced ectasia total deviation index (BAD-D) and stepwise logistic regression combined model (SLR2) in differentiating forme fruste keratoconus (FFKC) from normal. The area under the curve of TBI, F.Ele.Th, BAD-D and combined model SLR2 were 0.885, 0.874, 0.839 and 0.965, respectively

Table 3 shows the results from the CKC and FFKC groups compared to the control group for the ROC curve analysis, AUC, 95% confidence intervals, best cutoff point, sensitivity and specificity of the best cutoff points for each parameter, as well as the two combination models (SLR1 and SLR2). The majority of the observed parameters had sufficient strength (AUC > 0.80) to differentiate the CKC from the normal eyes, even the seven separate parameters and the SLR1 combined model had a discrimination efficiency of 100% (AUC = 1). Meanwhile, the overall predictive accuracy of these readings was moderate for eyes with FFKC (AUC < 0.80), and a single set of parameters, including bIOP, DA, A1V and A2T, failed to completely differentiate FFKC corneas from normal corneas. However, the SLR2 combined model (0.965) showed an excellent AUC, followed by TBI (0.885), F.Ele.Th (0.874) and BAD-D (0.839) (Fig. 1). Among the four AUC values mentioned above, except for SLR2 and F.Ele.Th (Z = 2.374, *p* = 0.0176, the DeLong test), there were no significant differences between any other two AUC values.

**Table 3:** Receiver operating characteristic curve analysis for ability of analyzed parameters to differentiate CKC and FFKC versus control eyes

	FFKC group versus control group				CKC group versus control group			
	AUC (95% CI)	Cutoff	Specificity, %	Sensitivity,%	AUC (95% CI)	Cutoff	Specificity, %	Sensitivity,%
*Stepwise logistic regression models*								
SLR1					1.000(0.962–1.000)	> 0	100.0	100.0
SLR2	0.965(0.887–0.995)	> 0.0956	84.0	100.0				
*Overall output parameters*								
TBI	0.885(0.781–0.951)	> 0.11	74.0	93.3	1.000(0.962–1.000)	> 0.91	100.0	100.0
F.Ele.Th (mm)	0.874(0.768–0.943)	> 3	88.0	66.7	0.978(0.924–0.997)	> 5	100.0	97.8
BAD-D	0.839(0.726–0.918)	> 1.73	90.0	80.0	1.000(0.962–1.000)	> 2.51	100.0	100.0
ISV	0.799(0.681–0.888)	> 18	64.0	93.3	1.000(0.962–1.000)	> 30	100.0	100.0
B.Ele.Th (mm)	0.783(0.664–0.876)	> 7	96.0	60.0	1.000(0.962–1.000)	> 11	100.0	100.0
ARTmax	0.772(0.651–0.867)	≤ 315	98.0	46.7	1.000(0.962–1.000)	≤ 284	100.0	100.0
IVA	0.760(0.638–0.857)	> 0.2	92.0	46.7	1.000(0.961–1.000)	> 0.25	98.0	100.0
KI	0.759(0.637–0.856)	> 1.04	78.0	60.0	0.977(0.923–0.997)	> 1.07	98.0	97.8
I–S (diopters)	0.755(0.632–0.853)	> 0.89	88.0	53.3	0.977(0.923–0.997)	> 1.42	98.0	97.8
CBI	0.752(0.629–0.851)	> 0.594	86.0	53.3	0.972(0.915–0.995)	> 0.891	96.0	93.3
TP (mm)	0.749(0.626–0.849)	≤ 516	78.0	66.7	0.975(0.920–0.996)	≤ 487	98.0	88.9
IHD	0.743(0.619–0.843)	> 0.016	86.0	60.0	1.000(0.962–1.000)	> 0.03	100.0	100.0
CCT (mm)	0.735(0.611–0.837)	≤ 531	60.0	80.0	0.942(0.874–0.979)	≤ 494	96.0	84.4
CH (mmHg)	0.728(0.603–0.831)	≤ 10.3	62.0	86.7	0.946(0.880–0.982)	≤ 8.9	98.0	77.8
SP-A1	0.716(0.591–0.821)	≤ 86.805	76.0	73.3	0.966(0.907–0.992)	≤ 68.892	94.0	88.9
CRF (mmHg)	0.709(0.583–0.815)	≤ 10.7	52.0	93.3	0.956(0.893–0.988)	≤ 8.5	96.0	88.9
ARTh	0.705(0.579–0.812)	≤ 454.163	44.0	93.3	0.953(0.889–0.986)	≤ 302.127	96.0	91.1
DA Ratio 2	0.687(0.560–0.796)	> 4.4722	72.0	66.7	0.943(0.875–0.980)	> 4.995	96.0	84.4
HC DfL	0.685(0.558–0.794)	≤ 6.776	64.0	73.3	0.748(0.649–0.832)	≤ 6.595	78.0	66.7
A1 DfA	0.675(0.547–0.786)	≤ 0.095	64.0	73.3	0.835(0.745–0.903)	> 0.104	92.0	66.7
DA Ratio 1	0.672(0.544–0.783)	> 1.5901	82.0	60.0	0.904(0.826–0.955)	> 1.617	92.0	88.9
Radius (mm)	0.665(0.537–0.778)	≤ 7.156	78.0	60.0	0.965(0.905–0.992)	≤ 6.613	98.0	91.1
CV 10 (mm3)	0.646(0.518–0.761)	≤ 60.2	60.0	66.7	0.793(0.698–0.869)	≤ 58.2	84.0	62.2
A2 DfA	0.641(0.513–0.757)	≤ 0.112	44.0	93.3	0.756(0.657–0.838)	> 0.121	86.0	57.8
CKI	0.639(0.511–0.755)	> 1.01	98.0	26.7	0.927(0.855–0.970)	> 1.01	98.0	91.1
A1 DfL	0.638(0.509–0.754)	≤ 2.392	44.0	86.7	0.584(0.478–0.684)	> 2.489	88.0	33.3
Astig F (diopters)	0.638(0.509–0.754)	≤ 1.3	44.0	80.0	0.850(0.762–0.915)	> 2.4	100.0	66.7
PD (mm)	0.631(0.503–0.748)	≤ 5.362	44.0	86.7	0.594(0.489–0.694)	≤ 5.405	38.0	86.7
A1T (ms)	0.610(0.481–0.729)	≤ 6.972	94.0	26.7	0.873(0.789–0.932)	≤ 7.018	94.0	75.6
Kmax F (diopters)	0.605(0.476–0.724)	> 44.8	62.0	60.0	0.990(0.944–1.000)	> 46.5	96.0	95.6
HCT (ms)	0.599(0.470–0.719)	> 16.632	22.0	93.3	0.571(0.465–0.672)	> 16.863	44.0	68.9
HC DfA	0.593(0.464–0.714)	≤ 0.964	52.0	73.3	0.812(0.719–0.885)	> 1.065	92.0	62.2
A2 DfL	0.561(0.433–0.684)	≤ 2.467	78.0	46.7	0.522(0.417–0.625)	≤ 3.239	40.0	75.6
IHA	0.555(0.427–0.679)	> 3.5	28.0	93.3	0.822(0.730–0.893)	> 15.3	94.0	71.1
Km F (diopters)	0.551(0.422–0.674)	> 43.9	70.0	53.3	0.889(0.808–0.944)	> 45.4	100.0	77.8
A2V (m/s)	0.542(0.414–0.666)	≤ − 0.275	34.0	86.7	0.863(0.777–0.925)	≤ − 0.309	82.0	82.2
A2T (ms)	0.525(0.397–0.650)	> 21.698	22.0	93.3	0.706(0.604–0.795)	> 22.098	68.0	71.1
A1V (m/s)	0.519(0.392–0.645)	≤ 0.179	14.0	100.0	0.768(0.670–0.848)	> 0.186	98.0	48.9
DA (mm)	0.519(0.392–0.645)	≤ 1.155	34.0	80.0	0.810(0.717–0.884)	> 1.216	88.0	66.7
bIOP (mmHg)	0.515(0.388–0.641)	> 14.1	44.0	66.7	0.716(0.614–0.804)	≤ 13.5	70.0	68.9

## Discussion

At present, early screening and diagnosis of keratoconus is one of the main concerns of cornea specialists and patients who are willing to undergo corneal refractive surgery [5, 14]. However, there are still some limitations with respect to the early detection of keratoconus. For instance, there are no visible clinical signs in the early stage of the disease, and even the majority of people only show a mild local corneal protuberance with normal corneal thickness [8, 10]. Therefore, it may not be useful for patients in the early stages of keratoconus or with no apparent signs to be assessed using only traditional morphological devices. In addition, although progressive thinning of keratoconus may be caused by weakened corneal biomechanical resistance and decreased biomechanical properties [4, 5], keratoconus cannot be solely attributed to changes in corneal morphology or biomechanical properties [19, 20].

Therefore, the primary purpose of this study was to comprehensively compare and analyze the influence of morphological and mechanical characteristics on the diagnosis of keratoconus using three different devices, the Pentacam (typical morphological measurement device), Corvis ST and ORA (two main in vivo biomechanical measurement devices), especially contralateral normal eye of unilateral keratoconus, that is, the forme fruste keratoconus, so as to obtain a more comprehensive diagnostic evaluation of early keratoconus.

In our study, the AUC of the device output parameters ranged from 0.515 to 0.885 between FFKC and normal eyes. Among the parameters measured in this study, the highest was recorded for TBI (0.885). The next eight parameters with higher AUC values (0.755–0.874) were all morphological parameters. To some extent, this might indicate that individual biomechanical parameters, except for TBI, had relatively weak diagnostic ability to distinguish forme fruste keratoconus.

However, it is noteworthy that, although the AUC of the deflection amplitude of the highest concavity (HC DfA) was 0.593, and there was no significant difference between the FFKC and the normal eyes, the SLR2 combined model obtained after stepwise logistic regression included the HC DfA. The SLR2 regression model had the highest AUC value of 0.965, with 100.0% sensitivity and 84.0% specificity. The SLR2 combination model also indicated that measurement of biomechanical parameters should not be considered as having no diagnostic capabilities for early keratoconus simply because there were no significant differences or the AUC values were small. Also, two biomechanical parameters, HC DfA and SP-A1, and one morphological parameter, F.Ele.Th, in the SLR2 regression model, again indicated the importance of considering biomechanics characteristics. Of course, biomechanical parameters need to be analyzed in combination with morphological parameters.

Based on the output parameters of the Pentacam and Corvis, Ambrósio et al. [21] introduced a new parameter, TBI, using random forest. This parameter was proved to be more accurate than all other independent parameter analyses used in the diagnosis of subclinical keratoconus. Most studies also found that TBI had a higher diagnostic ability to distinguish keratoconus [11], even in different stages of keratoconus [8]. In this study, TBI not only had the highest AUC value (CKC *vs*. normal, 1.000; FFKC *vs*. normal, 0.885), but it also was the only parameter that was included in the stepwise SLR1 regression model, which proved its effective diagnostic ability.

The first corneal stiffness value recorded in vivo by the Corvis ST, SP-A1, was developed by using displacement of the apex from the undeformed state to the first applanation in the deformation process. This measurement takes confounding factors into account, such as intraocular pressure and whole eye motion [22]. Some studies [13, 23] have suggested that reduced corneal biomechanical stability occurs prior to the alteration of corneal shape, and SP-A1 could be a potential biomarker to evaluate the progression of keratoconus. In our study, SP-A1 was the biomechanical parameter with the largest area under the ROC curve except for TBI and CBI, when distinguishing clinical keratoconus or forme fruste keratoconus (CKC vs. normal: 0.966; FFKC vs. normal: 0.716). These results demonstrated that the progress of keratoconus was related to the ability of the cornea to resist stress. In other words, corneal stiffness decreased with the continued progress of keratoconus, and thus, SP-A1 also decreased.

The anterior surface abnormality of keratoconus often appears earlier than the abnormalities in visual acuity and thickness [12]. Previous studies [24, 25] have suggested that anterior corneal elevation parameters are clinically relevant measures for detecting keratoconus and suspected cases of keratoconus. In our study that compared FFKC and normal eyes, among the 11 different morphological parameters that were assessed, five parameters appeared to fully characterize the different aspects of the asymmetry (height, curvature and others) of the anterior surface of the cornea. These five parameters were ISV, IVA, KI, IHD and I-S. Moreover, for the elevation of the front surface at the thinnest location, F.Ele.Th, its AUC value was the second largest when distinguishing FFKC from normal corneas. F.Ele.Th also was significant in the SLR2 regression model (*p* = 0.004).

According to the area under the ROC curve, both CH (CKC vs. normal, 0.946; FFKC vs. normal, 0.728) and CRF (CKC *vs*. normal, 0.956; FFKC vs. normal, 0.709) had medium discrimination abilities, and there were no differences in their diagnostic abilities (CKC vs. normal, *p* = 0.539; FFKC vs. normal, *p* = 0.805; the DeLong test). However, from the perspective of the regression model, when considering another in vivo biomechanical testing device, the function of the output parameters, CH and CRF, of the ORA were not reflected when the three devices were used to diagnose keratoconus or forme fruste keratoconus. Even when the ORA output was combined with the other two devices separately, CH and CRF were not included in the respective combined regression diagnosis models. This result was possibly because CH and CRF could only obtain the parameters at a certain point in the dynamic corneal process, but they could not dynamically reflect the entire corneal deformation process in real time [26, 27]. This result also suggested that the dynamic recording of the deformation process in real time was necessary to obtain parameters that indicated corneal biomechanical properties. However, these results need to be verified with a larger clinical sample.

Our study had certain limitations. First, only CH and CRF output parameters were included for ORA. The rest waveform parameters that might be associated with real-time corneal responses to air pulses were not included. Also, the sample size was relatively small, and hence, statistical analyses might need to be interpreted with caution. Therefore, it is necessary to provide additional analyzes using a larger sample size and more ORA parameters.

In conclusion, F.Ele.Th from Pentacam may be the most sensitive morphological parameter of forme fruste keratoconus, and the combination of F.Ele.Th, HC DfA and SP-A1 makes the diagnosis of FFKC more efficient. In addition to TBI, the SP-A1 output by Corvis ST also is worthy of attention. The CRF and CH output by ORA does not improve the combined diagnosis, despite the combination of corneal morphological and biomechanical properties that can optimize the diagnosis of forme fruste keratoconus. More ways to combine morphological and biomechanics characteristics in the future are worth exploring. Velocity at first application.

## Data Availability

Data can be shared upon request.

## Abbreviations

A1 DfA: The deflection amplitude of the first applanation
A1 DfL: The deflection length of the first applanation
A1T: The first applanation time
A1V: The first velocity of applanation
A2 DfA: The deflection amplitude of the second applanation
A2 DfL: The deflection length of the second applanation
A2T: The second applanation time
A2V: The second velocity of applanation
ARTh: Ambrósio relational thickness to the horizontal profile
ARTmax: Maximum Ambrósio relational thickness
Astig F: Central astigmatism from the anterior corneal surface
AUC: The area under the curve
B.Ele.Th: The elevation of the back surface at the thinnest location
BAD-D: Belin–Ambrósio enhanced ectasia total deviation index
bIOP: The biomechanical-corrected intraocular pressure
CBI: The Corvis biomechanical index
CCT: Corneal thickness at the apex of the cornea
CH: Corneal hysteresis
CKC: Clinical keratoconus
CKI: Central keratoconus index
Corvis ST: Corneal visualization Scheimpflug technology
CRF: Corneal resistance factor
CV 10: Corneal volume at 10 centered at the thinnest point
DA Ratio 1: The maximal value of the ratio between the deformation amplitude at the apex and at 1 mm from the corneal apex
DA Ratio 2: The maximal value of the ratio between the deformation amplitude at the apex and at 2 mm from the corneal apex
DA: Maximum deformation amplitude at the corneal apex
F.Ele.Th: The elevation of the front surface at the thinnest location
FFKC: Forme fruste keratoconus
HC DfA: The deflection amplitude of the highest concavity
HC DfL: The deflection length of the highest concavity
HCT: Time from the start until the highest concavity
IHA: Index of height asymmetry
IHD: Index of height decentration
I-S: Inferior–superior difference value
ISV: Index of surface variance
IVA: Index of vertical asymmetry
KI: Keratoconus index
Km F: Mean keratometry from the anterior corneal surface
Kmax F: Maximum keratometry from the anterior corneal surface
ORA: Ocular response analyzer
PD: Peak distance
QS: Quality specification
Radius: Central curvature radius at the highest concavity
ROC: Receiver operating characteristic
SD: Standard deviation
SLR1: The first stepwise logistic regression model
SLR2: The second stepwise logistic regression model
SP-A1: Stiffness parameter at the first applanation
TBI: The tomographic and biomechanical index
TKC: The topographic keratoconus classification
TP: Pachymetry at the thinnest point

## Code availability

Code can be shared upon request.
